# Dichlorido[1-(1,10-phenanthrolin-2-yl)-2-pyridone]copper(II)

**DOI:** 10.1107/S160053680801948X

**Published:** 2008-07-05

**Authors:** Jin Min Li

**Affiliations:** a Chemistry and Chemical Engineering College, Shanxi Datong University, Datong 037008, People’s Republic of China

## Abstract

In the title mononuclear complex, [CuCl_2_(C_17_H_11_N_3_O)], the Cu^II^ ion is in a distorted square-pyramidal coordination environment. The crystal structure is stabilized by various π–π stacking inter­actions in which the benzene ring, a pyridine ring and the five-membered CuN_2_C_2_ ring are involved. The centroid–centroid distances range from 3.5631 (15) to 3.5666 (16) Å.

## Related literature

For a related structure, see: Liu *et al.* (2008 ▶).
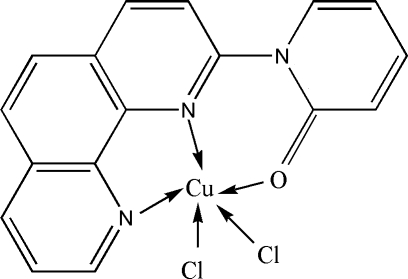


## Experimental

### 

#### Crystal data

[CuCl_2_(C_17_H_11_N_3_O)]
*M* *_r_* = 407.73
Monoclinic, 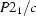

*a* = 7.3653 (12) Å
*b* = 13.811 (2) Å
*c* = 14.994 (2) Å
β = 98.416 (2)°
*V* = 1508.8 (4) Å^3^
*Z* = 4
Mo *K*α radiation
μ = 1.81 mm^−1^
*T* = 298 (2) K
0.38 × 0.16 × 0.12 mm

#### Data collection

Bruker SMART APEX CCD diffractometer
Absorption correction: multi-scan (*SADABS*; Sheldrick, 1996 ▶) *T*_min_ = 0.546, *T*_max_ = 0.812
8630 measured reflections
3265 independent reflections
2706 reflections with *I* > 2σ(*I*)
*R*_int_ = 0.035

#### Refinement

*R*[*F* ^2^ > 2σ(*F* ^2^)] = 0.037
*wR*(*F* ^2^) = 0.102
*S* = 1.11
3265 reflections
217 parameters
1 restraint
H-atom parameters constrained
Δρ_max_ = 0.64 e Å^−3^
Δρ_min_ = −0.66 e Å^−3^

### 

Data collection: *SMART* (Bruker, 1997 ▶); cell refinement: *SAINT* (Bruker, 1997 ▶); data reduction: *SAINT*; program(s) used to solve structure: *SHELXTL* (Sheldrick, 2008 ▶); program(s) used to refine structure: *SHELXTL*; molecular graphics: *SHELXTL*; software used to prepare material for publication: *SHELXTL*.

## Supplementary Material

Crystal structure: contains datablocks I, global. DOI: 10.1107/S160053680801948X/lh2647sup1.cif

Structure factors: contains datablocks I. DOI: 10.1107/S160053680801948X/lh2647Isup2.hkl

Additional supplementary materials:  crystallographic information; 3D view; checkCIF report

## Figures and Tables

**Table:** 

Cl1—Cu1	2.2362 (8)
Cl2—Cu1	2.3740 (8)
Cu1—N2	2.0165 (19)
Cu1—N1	2.042 (2)
Cu1—O1	2.1337 (18)

**Table:** 

N2—Cu1—N1	80.75 (8)
N2—Cu1—O1	80.70 (7)
N1—Cu1—O1	147.25 (8)
N2—Cu1—Cl1	158.03 (7)
N1—Cu1—Cl1	92.93 (6)
O1—Cu1—Cl1	94.29 (5)
N2—Cu1—Cl2	97.87 (6)
N1—Cu1—Cl2	115.81 (6)
O1—Cu1—Cl2	93.32 (6)
Cl1—Cu1—Cl2	103.79 (3)

**Fig. 1.**
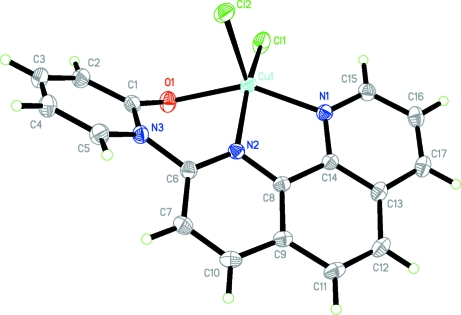
The molecular structure of the title complex with atom-numbering scheme. Displacement ellipsoids are shown at the 30% probability level

## supplementary crystallographic information

### Comment

Derivatives of 1,10-phenanthroline play an important role in modern coordination chemistry and the complex with 1-(1,10-phenanthrolin-2-yl)-2-pyridone as bridging ligand and termial ligand has already been reported (Liu *et al.*, 2008). Herein the crystal structure of the title complex with 1-(1,10-phenanthrolin-2-yl)-2-pyridone as terminal ligand is reported.

Fig. 1 shows the moleculuar structure, revealing that the atom Cu^II^ ion is in a distorted square-pyramidal coordination environment, with atom Cl2 in the apical position. There are π–π stacking interactions involving symmetry-related complex molecules, the relevant distances being *Cg*1···*Cg*2^i^ = 3.5631 (15) Å and *Cg*1···*Cg*2^i^_perp_ = 3.355 Å and α = 4.35°; *Cg*2···*Cg*3^ii^ = 3.5568 (16) Å and *Cg*2···*Cg*3^ii^_perp_ = 3.450 Å and α = 1.81°; *Cg*2···*Cg*2^i^ = 3.5666 (16) Å and *Cg*2···*Cg*2^i^_perp_ = 3.407 Å and α = 0.00° [symmetry codes: (i) 1-*x*, 2-*x*, 1-*x*; (ii) -*x*, 2-*y*, 1-*x*; *Cg*1, *Cg*2 and *Cg*3 are the centroids of the Cu1/N1/N2/C8/C14 ring, C8/C9/C11-C14 ring amd N1/C13-C17 ring, respectively; *Cg*i···*Cg*j_perp_ is the perpendicular distance from ring *Cg*i to ring *Cg*j; α is the dihedral angle between ring plane *Cg*i and ring plane *Cg*j]. These π–π stacking interactions help stabilize the crystal structure.

### Experimental

A 10 ml methanol solution of 1-(1,10-phenanthrolin-2-yl)-2-pyridone (0.1648 g, 0.603 mmol) was added into a 10 ml methanol solution containing CuCl_2_ (0.1025 g, 0.601 mmol) and the mixture was stirred for a few minutes. The green single crystals were obtained after the filtrate had been allowed to stand at room temperature for two weeks.

### Refinement

All H atoms were placed in calculated positions with C—H = 0.93 Å and refined as riding with *U*_iso_(H) = 1.2*U*_eq_(C).

### Crystal data

**Table:**

[CuCl_2_(C_17_H_11_N_3_O)]	*F*_000_ = 820
*M**_r_* = 407.73	*D*_x_ = 1.795 Mg m^−^^3^
Monoclinic, *P*2_1_/*c*	Mo *K*α radiation λ = 0.71073 Å
Hall symbol: -P 2ybc	Cell parameters from 3380 reflections
*a* = 7.3653 (12) Å	θ = 2.8–28.1º
*b* = 13.811 (2) Å	µ = 1.81 mm^−^^1^
*c* = 14.994 (2) Å	*T* = 298 (2) K
β = 98.416 (2)º	Block, green
*V* = 1508.8 (4) Å^3^	0.38 × 0.16 × 0.12 mm
*Z* = 4	

### Data collection

**Table:**

Bruker SMART APEX CCD diffractometer	3265 independent reflections
Radiation source: fine-focus sealed tube	2706 reflections with *I* > 2σ(*I*)
Monochromator: graphite	*R*_int_ = 0.035
*T* = 298(2) K	θ_max_ = 27.0º
φ and ω scans	θ_min_ = 2.0º
Absorption correction: multi-scan(SADABS; Sheldrick, 1996)	*h* = −9→9
*T*_min_ = 0.546, *T*_max_ = 0.812	*k* = −16→17
8630 measured reflections	*l* = −16→19

### Refinement

**Table:**

Refinement on *F*^2^	Secondary atom site location: difference Fourier map
Least-squares matrix: full	Hydrogen site location: inferred from neighbouring sites
*R*[*F*^2^ > 2σ(*F*^2^)] = 0.037	H-atom parameters constrained
*wR*(*F*^2^) = 0.102	*w* = 1/[σ^2^(*F*_o_^2^) + (0.0547*P*)^2^ + 0.1919*P*] where *P* = (*F*_o_^2^ + 2*F*_c_^2^)/3
*S* = 1.11	(Δ/σ)_max_ = 0.002
3265 reflections	Δρ_max_ = 0.64 e Å^−^^3^
217 parameters	Δρ_min_ = −0.66 e Å^−^^3^
1 restraint	Extinction correction: none
Primary atom site location: structure-invariant direct methods	

### Special details

**Table:**

Geometry. All e.s.d.'s (except the e.s.d. in the dihedral angle between two l.s. planes) are estimated using the full covariance matrix. The cell e.s.d.'s are taken into account individually in the estimation of e.s.d.'s in distances, angles and torsion angles; correlations between e.s.d.'s in cell parameters are only used when they are defined by crystal symmetry. An approximate (isotropic) treatment of cell e.s.d.'s is used for estimating e.s.d.'s involving l.s. planes.
Refinement. Refinement of *F*^2^ against ALL reflections. The weighted *R*-factor *wR* and goodness of fit *S* are based on *F*^2^, conventional *R*-factors *R* are based on *F*, with *F* set to zero for negative *F*^2^. The threshold expression of *F*^2^ > σ(*F*^2^) is used only for calculating *R*-factors(gt) *etc*. and is not relevant to the choice of reflections for refinement. *R*-factors based on *F*^2^ are statistically about twice as large as those based on *F*, and *R*- factors based on ALL data will be even larger.

### Fractional atomic coordinates and isotropic or equivalent isotropic displacement parameters (Å^2^)

**Table:**

	*x*	*y*	*z*	*U*_iso_*/*U*_eq_	
C1	0.3230 (4)	1.05721 (18)	0.15163 (16)	0.0310 (5)	
C2	0.3160 (4)	1.0755 (2)	0.05804 (17)	0.0378 (6)	
H2	0.3550	1.1354	0.0397	0.045*	
C3	0.2546 (4)	1.0089 (2)	−0.00543 (17)	0.0397 (6)	
H3	0.2518	1.0236	−0.0662	0.048*	
C4	0.1951 (4)	0.9179 (2)	0.01991 (18)	0.0405 (6)	
H4	0.1498	0.8725	−0.0236	0.049*	
C5	0.2045 (4)	0.89731 (19)	0.10802 (18)	0.0371 (6)	
H5	0.1638	0.8373	0.1252	0.045*	
C6	0.2990 (3)	0.93054 (17)	0.26475 (16)	0.0291 (5)	
C7	0.3620 (4)	0.83639 (18)	0.28418 (18)	0.0352 (6)	
H7	0.3909	0.7963	0.2385	0.042*	
C8	0.2832 (3)	0.95921 (16)	0.41397 (15)	0.0254 (5)	
C9	0.3410 (3)	0.86537 (16)	0.43983 (17)	0.0289 (5)	
C10	0.3804 (4)	0.80403 (18)	0.37072 (19)	0.0358 (6)	
H10	0.4194	0.7409	0.3841	0.043*	
C11	0.3575 (4)	0.83935 (19)	0.53288 (18)	0.0354 (6)	
H11	0.4002	0.7780	0.5510	0.042*	
C12	0.3121 (4)	0.9024 (2)	0.59522 (17)	0.0361 (6)	
H12	0.3233	0.8835	0.6553	0.043*	
C13	0.2472 (3)	0.99763 (18)	0.57023 (16)	0.0308 (5)	
C14	0.2373 (3)	1.02632 (17)	0.47986 (15)	0.0258 (5)	
C15	0.1357 (4)	1.17865 (19)	0.50840 (17)	0.0339 (6)	
H15	0.0987	1.2402	0.4882	0.041*	
C16	0.1378 (4)	1.1564 (2)	0.59914 (18)	0.0389 (6)	
H16	0.1007	1.2022	0.6382	0.047*	
C17	0.1950 (4)	1.06686 (19)	0.63025 (17)	0.0362 (6)	
H17	0.1993	1.0518	0.6910	0.043*	
Cl1	0.25172 (13)	1.29316 (5)	0.33181 (5)	0.0508 (2)	
Cl2	−0.07342 (10)	1.12202 (5)	0.21487 (5)	0.04360 (19)	
Cu1	0.20539 (5)	1.13365 (2)	0.316143 (19)	0.03221 (13)	
N1	0.1845 (3)	1.11538 (14)	0.44938 (13)	0.0282 (4)	
N2	0.2618 (3)	0.99094 (14)	0.32770 (12)	0.0262 (4)	
N3	0.2737 (3)	0.96350 (15)	0.17385 (13)	0.0299 (5)	
O1	0.3712 (3)	1.11913 (12)	0.21153 (13)	0.0375 (4)	

### Atomic displacement parameters (Å^2^)

**Table:**

	*U*^11^	*U*^22^	*U*^33^	*U*^12^	*U*^13^	*U*^23^
C1	0.0304 (14)	0.0318 (13)	0.0311 (13)	0.0018 (10)	0.0054 (10)	−0.0030 (11)
C2	0.0418 (17)	0.0382 (15)	0.0345 (14)	−0.0002 (12)	0.0091 (12)	0.0034 (11)
C3	0.0423 (17)	0.0498 (16)	0.0270 (13)	0.0047 (13)	0.0049 (11)	−0.0005 (12)
C4	0.0477 (18)	0.0415 (15)	0.0310 (14)	−0.0004 (13)	0.0012 (12)	−0.0108 (11)
C5	0.0443 (17)	0.0306 (13)	0.0353 (14)	−0.0014 (12)	0.0018 (12)	−0.0057 (11)
C6	0.0308 (14)	0.0287 (12)	0.0279 (12)	−0.0011 (10)	0.0043 (10)	−0.0021 (10)
C7	0.0404 (16)	0.0271 (12)	0.0387 (14)	0.0034 (11)	0.0072 (12)	−0.0045 (11)
C8	0.0208 (13)	0.0263 (11)	0.0284 (12)	−0.0017 (9)	0.0009 (9)	0.0023 (9)
C9	0.0239 (14)	0.0275 (12)	0.0348 (13)	−0.0011 (9)	0.0025 (10)	0.0048 (9)
C10	0.0350 (16)	0.0263 (13)	0.0448 (15)	0.0032 (11)	0.0009 (11)	0.0033 (11)
C11	0.0306 (15)	0.0332 (13)	0.0409 (15)	−0.0015 (11)	0.0002 (11)	0.0134 (11)
C12	0.0358 (16)	0.0427 (14)	0.0287 (13)	−0.0075 (12)	0.0011 (11)	0.0111 (11)
C13	0.0242 (14)	0.0394 (13)	0.0287 (12)	−0.0097 (11)	0.0030 (10)	0.0034 (11)
C14	0.0199 (12)	0.0300 (12)	0.0266 (12)	−0.0030 (9)	0.0007 (9)	0.0013 (9)
C15	0.0366 (15)	0.0319 (13)	0.0330 (13)	0.0003 (11)	0.0041 (11)	−0.0058 (11)
C16	0.0396 (17)	0.0454 (15)	0.0328 (14)	−0.0039 (12)	0.0093 (12)	−0.0096 (12)
C17	0.0349 (16)	0.0472 (16)	0.0266 (13)	−0.0104 (12)	0.0050 (11)	−0.0015 (11)
Cl1	0.0949 (7)	0.0249 (3)	0.0346 (4)	0.0034 (3)	0.0158 (4)	0.0007 (3)
Cl2	0.0389 (4)	0.0562 (4)	0.0337 (4)	0.0037 (3)	−0.0013 (3)	0.0073 (3)
Cu1	0.0477 (3)	0.02408 (19)	0.02382 (19)	0.00415 (13)	0.00175 (14)	0.00028 (11)
N1	0.0279 (12)	0.0295 (10)	0.0264 (10)	−0.0004 (8)	0.0018 (8)	−0.0009 (8)
N2	0.0284 (11)	0.0228 (10)	0.0271 (10)	0.0009 (8)	0.0033 (8)	−0.0002 (8)
N3	0.0362 (13)	0.0280 (11)	0.0263 (10)	0.0009 (9)	0.0066 (9)	−0.0024 (8)
O1	0.0475 (12)	0.0328 (10)	0.0335 (10)	−0.0075 (8)	0.0104 (8)	−0.0047 (8)

### Geometric parameters (Å, °)

**Table:**

C1—O1	1.253 (3)	C9—C11	1.429 (4)
C1—N3	1.397 (3)	C10—H10	0.9300
C1—C2	1.419 (3)	C11—C12	1.355 (4)
C2—C3	1.353 (4)	C11—H11	0.9300
C2—H2	0.9300	C12—C13	1.430 (4)
C3—C4	1.402 (4)	C12—H12	0.9300
C3—H3	0.9300	C13—C14	1.403 (3)
C4—C5	1.343 (4)	C13—C17	1.405 (4)
C4—H4	0.9300	C14—N1	1.349 (3)
C5—N3	1.386 (3)	C15—N1	1.330 (3)
C5—H5	0.9300	C15—C16	1.393 (4)
C6—N2	1.318 (3)	C15—H15	0.9300
C6—C7	1.397 (3)	C16—C17	1.366 (4)
C6—N3	1.423 (3)	C16—H16	0.9300
C7—C10	1.360 (4)	C17—H17	0.9300
C7—H7	0.9300	Cl1—Cu1	2.2362 (8)
C8—N2	1.353 (3)	Cl2—Cu1	2.3740 (8)
C8—C9	1.401 (3)	Cu1—N2	2.0165 (19)
C8—C14	1.431 (3)	Cu1—N1	2.042 (2)
C9—C10	1.401 (4)	Cu1—O1	2.1337 (18)
			
O1—C1—N3	121.2 (2)	C14—C13—C17	116.5 (2)
O1—C1—C2	123.5 (2)	C14—C13—C12	118.7 (2)
N3—C1—C2	115.3 (2)	C17—C13—C12	124.8 (2)
C3—C2—C1	122.3 (3)	N1—C14—C13	123.8 (2)
C3—C2—H2	118.8	N1—C14—C8	116.2 (2)
C1—C2—H2	118.8	C13—C14—C8	120.0 (2)
C2—C3—C4	120.3 (2)	N1—C15—C16	122.7 (2)
C2—C3—H3	119.9	N1—C15—H15	118.7
C4—C3—H3	119.9	C16—C15—H15	118.7
C5—C4—C3	118.9 (3)	C17—C16—C15	119.5 (2)
C5—C4—H4	120.6	C17—C16—H16	120.3
C3—C4—H4	120.6	C15—C16—H16	120.3
C4—C5—N3	121.4 (3)	C16—C17—C13	119.8 (2)
C4—C5—H5	119.3	C16—C17—H17	120.1
N3—C5—H5	119.3	C13—C17—H17	120.1
N2—C6—C7	122.5 (2)	N2—Cu1—N1	80.75 (8)
N2—C6—N3	118.1 (2)	N2—Cu1—O1	80.70 (7)
C7—C6—N3	119.4 (2)	N1—Cu1—O1	147.25 (8)
C10—C7—C6	119.2 (2)	N2—Cu1—Cl1	158.03 (7)
C10—C7—H7	120.4	N1—Cu1—Cl1	92.93 (6)
C6—C7—H7	120.4	O1—Cu1—Cl1	94.29 (5)
N2—C8—C9	123.5 (2)	N2—Cu1—Cl2	97.87 (6)
N2—C8—C14	116.4 (2)	N1—Cu1—Cl2	115.81 (6)
C9—C8—C14	120.1 (2)	O1—Cu1—Cl2	93.32 (6)
C8—C9—C10	116.2 (2)	Cl1—Cu1—Cl2	103.79 (3)
C8—C9—C11	118.8 (2)	C15—N1—C14	117.8 (2)
C10—C9—C11	125.0 (2)	C15—N1—Cu1	129.52 (17)
C7—C10—C9	120.3 (2)	C14—N1—Cu1	112.63 (15)
C7—C10—H10	119.9	C6—N2—C8	118.2 (2)
C9—C10—H10	119.9	C6—N2—Cu1	128.23 (16)
C12—C11—C9	121.2 (2)	C8—N2—Cu1	113.10 (15)
C12—C11—H11	119.4	C5—N3—C1	121.5 (2)
C9—C11—H11	119.4	C5—N3—C6	117.0 (2)
C11—C12—C13	121.1 (2)	C1—N3—C6	121.5 (2)
C11—C12—H12	119.4	C1—O1—Cu1	117.28 (17)
C13—C12—H12	119.4		
			
O1—C1—C2—C3	−176.6 (3)	Cl1—Cu1—N1—C15	25.1 (2)
N3—C1—C2—C3	4.1 (4)	Cl2—Cu1—N1—C15	−81.8 (2)
C1—C2—C3—C4	−0.1 (4)	N2—Cu1—N1—C14	7.26 (17)
C2—C3—C4—C5	−1.7 (4)	O1—Cu1—N1—C14	−49.0 (2)
C3—C4—C5—N3	−0.8 (4)	Cl1—Cu1—N1—C14	−151.57 (16)
N2—C6—C7—C10	2.2 (4)	Cl2—Cu1—N1—C14	101.59 (16)
N3—C6—C7—C10	−177.9 (2)	C7—C6—N2—C8	−1.1 (4)
N2—C8—C9—C10	1.1 (4)	N3—C6—N2—C8	179.0 (2)
C14—C8—C9—C10	179.1 (2)	C7—C6—N2—Cu1	170.42 (19)
N2—C8—C9—C11	−179.5 (2)	N3—C6—N2—Cu1	−9.5 (3)
C14—C8—C9—C11	−1.6 (3)	C9—C8—N2—C6	−0.6 (4)
C6—C7—C10—C9	−1.6 (4)	C14—C8—N2—C6	−178.6 (2)
C8—C9—C10—C7	0.0 (4)	C9—C8—N2—Cu1	−173.35 (19)
C11—C9—C10—C7	−179.3 (3)	C14—C8—N2—Cu1	8.6 (3)
C8—C9—C11—C12	2.4 (4)	N1—Cu1—N2—C6	179.5 (2)
C10—C9—C11—C12	−178.3 (3)	O1—Cu1—N2—C6	−27.6 (2)
C9—C11—C12—C13	−0.5 (4)	Cl1—Cu1—N2—C6	−105.8 (2)
C11—C12—C13—C14	−2.2 (4)	Cl2—Cu1—N2—C6	64.5 (2)
C11—C12—C13—C17	179.1 (3)	N1—Cu1—N2—C8	−8.60 (16)
C17—C13—C14—N1	1.2 (4)	O1—Cu1—N2—C8	144.29 (17)
C12—C13—C14—N1	−177.6 (2)	Cl1—Cu1—N2—C8	66.0 (2)
C17—C13—C14—C8	−178.3 (2)	Cl2—Cu1—N2—C8	−123.61 (16)
C12—C13—C14—C8	3.0 (3)	C4—C5—N3—C1	5.1 (4)
N2—C8—C14—N1	−2.5 (3)	C4—C5—N3—C6	−172.4 (3)
C9—C8—C14—N1	179.4 (2)	O1—C1—N3—C5	174.1 (2)
N2—C8—C14—C13	177.0 (2)	C2—C1—N3—C5	−6.5 (3)
C9—C8—C14—C13	−1.1 (3)	O1—C1—N3—C6	−8.4 (4)
N1—C15—C16—C17	1.2 (4)	C2—C1—N3—C6	170.9 (2)
C15—C16—C17—C13	−1.3 (4)	N2—C6—N3—C5	−142.1 (2)
C14—C13—C17—C16	0.2 (4)	C7—C6—N3—C5	38.0 (3)
C12—C13—C17—C16	178.9 (3)	N2—C6—N3—C1	40.3 (3)
C16—C15—N1—C14	0.2 (4)	C7—C6—N3—C1	−139.6 (3)
C16—C15—N1—Cu1	−176.3 (2)	N3—C1—O1—Cu1	−46.7 (3)
C13—C14—N1—C15	−1.4 (4)	C2—C1—O1—Cu1	134.1 (2)
C8—C14—N1—C15	178.1 (2)	N2—Cu1—O1—C1	55.40 (19)
C13—C14—N1—Cu1	175.69 (18)	N1—Cu1—O1—C1	111.6 (2)
C8—C14—N1—Cu1	−4.8 (3)	Cl1—Cu1—O1—C1	−146.15 (19)
N2—Cu1—N1—C15	−176.1 (2)	Cl2—Cu1—O1—C1	−42.04 (19)
O1—Cu1—N1—C15	127.7 (2)		
