# 2-Carboxy­pyridinium hydrogen chloranilate. Corrigendum

**DOI:** 10.1107/S1600536809004528

**Published:** 2009-02-13

**Authors:** Youhei Tabuchi, Akiko Takahashi, Kazuma Gotoh, Haruo Akashi, Hiroyuki Ishida

**Affiliations:** a Department of Chemistry, Faculty of Science, Okayama University, Okayama 700-8530, Japan; b Research Institute of Natural Sciences, Okayama University of Science, Okayama 700-0005, Japan

## Abstract

The title and the chemical names of the paper by Tabuchi, Takahashi, Gotoh, Akashi & Ishida [*Acta Cryst.* (2005), E**61**, o4215–o4217] are corrected.

## Abstract 2

Corrigendum to *Acta Cryst.* (2005), E**61**, o4215–o4217.

In the paper by Tabuchi, Takahashi, Gotoh, Akashi & Ishida [*Acta Cryst.* (2005), E**61**, o4215–o4217], the title and the chemical names are incorrect with regard to the position of the carboxy group. The correct title of the original paper should be ‘3-Carboxypyridinium hydrogen chloranilate’ and the chemical names 2-carboxypyridine and 2-carboxypyridinium in the *Abstract* should be 3-carboxypyridine and 3-carboxy­pyridinium, respectively.

